# Histomorphological and Redox Delineations in the Testis and Epididymis of Albino Rats Fed with Green-Synthesized Cellulose

**DOI:** 10.3390/biology9090246

**Published:** 2020-08-25

**Authors:** Chiagoziem A. Otuechere, Adewale Adewuyi, Olusegun L. Adebayo, Emmanuel Yawson, Omolara Kabiawu, Sarah Al-Rashed, Blessing Okubio, Amany M. Beshbishy, Gaber El-Saber Batiha

**Affiliations:** 1 Department of Biochemistry, Redeemer’s University, Ede, Osun State 232102, Nigeria; adebayool@run.edu.ng (O.L.A.); kabiawuomolara@yahoo.com (O.K.); okubiob@run.edu.ng (B.O.); 2 Department of Chemical Sciences, Redeemer’s University, Ede, Osun State 232102, Nigeria; walexy62@yahoo.com; 3 Department of Anatomy, Redeemer’s University, Ede, Osun State 232102, Nigeria; yawsone@run.edu.ng; 4 Department of Botany and Microbiology, College of Science, King Saud University, Riyadh 11451, Saudi Arabia; salrashed@ksu.edu.sa; 5 National Research Center for Protozoan Disease, Obihiro University of Agriculture and Veterinary Medicine, Nishi 2-13, Inada-cho, Obihiro 080-8555, Hokkaido, Japan; amanimagdi2008@gmail.com; 6 Department of Pharmacology and Therapeutics, Faculty of Veterinary Medicine, Damanhour University, Damanhour, AlBeheira 22511, Egypt; gaberbatiha@gmail.com

**Keywords:** green-synthesized cellulose, redox status, histomorphology, testes, epididymis

## Abstract

It has also become increasingly necessary to diversify the production of cellulose for biomedical applications. In this study, cellulose-green-synthesized from *Sesamum indicum* (GSC)—was administered orally to rats for 14 days as follows: control, 100, 200 and 400 mg/kg GSC. The impact of GSC on the antioxidant status and histomorphology of the testes and epididymis were studied. GSC had no effects on organ weights and organosomatic indices. In the testes, GSC caused nonsignificant changes in superoxide dismutase, catalase, reduced glutathione and nitric oxide levels, whereas it significantly decreased glutathione peroxidase and malondialdehyde levels. In the epididymis, GSC significantly decreased superoxide dismutase and nitric oxide levels, but caused a significant increase in glutathione peroxidase and reduced glutathione levels. Furthermore, at ×200 magnification, testicular morphology appeared normal at all doses, however, extravasation of the germinal epithelium of the epididymis was observed at doses of 200 and 400 mg/kg GSC. Conversely, at ×400 magnification, spermatogenic arrest (testes) and chromatolytic alterations (epididymis) were observed at the higher doses (200 and 400 mg/kg GSC). This study reports on the effect of green-synthesized cellulose on testicular and epididymal histology and redox status and further extends the frontiers of research on cellulose.

## 1. Introduction

Cellulose and its derivatives have been identified as part of the group of isolated or synthetic nondigestible carbohydrates that could be designated as “generally regarded as safe” (GRAS) and used as a food additive [1]. Within the last few years, interest in the use of cellulose has heightened. Cellulose has variously been added in soda drinks, sauces, ice-cream toppings and cheese products. Cellulose also reinforces the dietary needs of some ruminants and termites, while in humans, it acts as a bulking substance that aids bowel movement. The utility of cellulose in the textile and paper industries have long been recognized [2].

Not surprisingly, various contending applications have seen conventional sources of cellulose become scarce and expensive. Furthermore, natural cellulose is thermally unstable, incompatible with hydrophobic polymers and has high moisture retention. These constraints have raised the need to seek for nonconventional and inexpensive substitutes [3,4]. In our laboratories, we had previously converted available cellulose into nanocellulose [5,6] or blended it with vermiculite polymer to yield composites with enhanced materiality [7]. The green synthesis approach, exploiting plant extracts as reducing agents, offer cost—and eco-friendly alternatives to the physical and chemical methods of synthesis. Several studies have reported the synthesis of cellulose using jackfruit leaves [8], *Retama raetam* [9] and *Citrullus lanatus* [10] as capping agent. In this present study, cellulose was synthesized from *Sesamum indicum*, an oilseed crop cultivated in Nigeria.

A sub-chronic study in rats fed with 5-g/kg hydroxypropyl methylcellulose reported a non-observed-adverse-effect level [11]. In yet another study, it was shown that patients suffering from diarrhea and constipation showed no adverse effects during treatment with an oral dose of modified cellulose, up to 6 g per day over a period of 32 weeks [12]. Although cellulose is generally believed to be nontoxic, cellulose modified from watermelon exocarp produced histological abnormalities in rats following 14 days of oral ingestion [10]. Previous toxicological studies in the testes of mice exposed to cellulose nanocrystals via inhalation, also revealed elevation in oxidative stress parameters and damage to testicular structure [13]. Histopathological changes were widely used as biomarkers in toxicity assessment because alterations found in target organs are easily delineated and serve as warning signs of impairment [14]. Moreover, considering the widespread exposure of humans to cellulose and the limited data on its effect on male reproductive organs, the present study explored whether green-synthesized cellulose distorted redox homeostasis and morphology in the testes and epididymis of male Wistar rats.

## 2. Materials and Methods

### 2.1. Chemicals

Epinephrine, thiobarbituric acid, reduced glutathione, trichloroacetic acid and dithio-bis-2-nitrobenzoic acid were purchased from Sigma Chemicals (St. Loius, MO, USA). Analytical grade reagents from reputable vendors were used.

### 2.2. Synthesis of GSC

The synthesis of cellulose from *Sesamum indicum* seeds, it is chemical modification and characterization are as previously described [3]. Briefly, 200 g of powdered *Sesamum indicum* was subjected to heat and mechanical stirring in alkali for 5 h. After a series of washing to remove the alkali, the residue was bleached in equal volumes of acetate buffer and sodium chlorite and subjected to another round of heating at 80 °C for 5 h to produce fibers. The resulting fibers were bleached repeatedly until completely white. Further modifications involved mixing the fiber with chloroacetylchloride (minus excess thionyl chloride) and subjecting the mixture to 80 °C heating for 5 h. After cooling in ice, 50 mL suberic acid (50 mL) was added to the mixture followed by continuous stirring and repeated centrifugation for 10 min at 8500× *g* to remove excess suberic acid. The final product was dried at 50 °C for 24 h giving rise to green-synthesized cellulose (GSC). Fourier-transform infrared spectroscopy analyses revealed characteristic amorphous and β-1,4-glycosidic linkage bands at 354 cm^−1^ and 894 cm^−1^, respectively. Moreover, crystallinity and particle size distribution of GSC was 77.03% and 10.3 µm, respectively.

### 2.3. Animal Model and Experimental Design

Twenty sexually mature male Wistar rats (193 ± 15 g) nurtured at the Redeemer’s University, Animal House Facility, Ede, Nigeria, were used for this study. Animals were kept in a well-aerated room and fed with rat chow and water ad libitum. The study approval was coded RUN/BCH/12/4601 and was conducted following the guidelines outlined by the Redeemer’s University Research Ethics on Animal Research. Animals were grouped into four (n = 5). Sample size was determined using the law of diminishing returns as previously proposed [15]. Applying the formula: *Degree of freedom of analysis of variance = Total number of animals—Total number of groups*, the number 16 was obtained, which falls within the recommended sample size of between 10 and 20. Group A rats received normal saline and served as the control, while rats assigned to groups B–D were fed with 100 mg/kg, 200 mg/kg and 400 mg/kg GSC, respectively, per *os*, for 14 days. Our earlier report [10] informed the dose selection for GSC. Twenty-four hours after the last administration of GSC, the animals were sacrificed after mild ether anesthetic. The testes and epididymis were removed and weighed. The antioxidant enzymes and oxidative stress markers were evaluated using testicular and epididymal post-mitochondrial fractions.

### 2.4. Biochemical Assays

Testicular and epididymal total protein concentrations were determined based on the method of Gornall et al. [16], while the superoxide dismutase (SOD) activity was determined in a mixture of sample, epinephrine and sodium carbonate buffer at the wavelength of 480 nm [17]. Catalase (CAT) was assayed using hydrogen peroxide as a substrate in the presence of 0.01-M phosphate buffer [18]. Glutathione peroxidase (GPx) and reduced glutathione (GSH) levels were determined using 5, 5′-dithiobis (2-nitrobenzoic) acid as substrate as previously described [19,20]. Nitric oxide (NO) level was determined by the Griess reaction, according to Bryan and Grisham [21] while lipid peroxidation, quantified as malondialdehyde (MDA), was determined in the reaction mixture containing thiobarbituric acid and trichloroacetic acid, which was incubated at 80 °C and measurement obtained at 532 nm [22].

### 2.5. Histological Studies

Testis and epididymis were fixed with Bouin’s solution and 10% buffered formalin. Fixed specimen was processed at the Histopathology unit at the University College Hospital, University of Ibadan, Ibadan, Nigeria. After embedding tissues in paraffin wax, sections were processed using hematoxylin and eosin. Stained sections were analyzed using the Amscope microscope camera attached to an electrical light microscope. Organs were imaged throughout the depth and length at magnifications of ×200 and ×400 to visualize morphologic structures.

### 2.6. Statistics

Data were denoted as the mean ± standard error of the mean (SEM). Normality (Shapiro-Wilk test) and homoscedasticity (Brown-Forsythe test) were tested prior to further analysis. Intergroup differences were determined by one-way analysis of variance (ANOVA), followed by Dunnett’s multiple comparison post hoc test (GraphPad Prism software version 8, Inc., San Diego, CA, USA). Significance was taken at *p <* 0.05.

## 3. Results

### 3.1. Effect of GSC on Testicular and Epididymal Absolute Weights, Organosomatic Indices and Protein Levels

The absolute weights, organosomatic indices and protein levels of the rats are presented in Table 1. GSC administered at the doses of 100, 200 and 400 mg/kg did not produce any effect on the absolute weights, organosomatic indices and total proteins of testes and epididymis of the treated rats.

### 3.2. Effect of GSC on Testicular and Epididymal Redox Status

**Figure 1:**
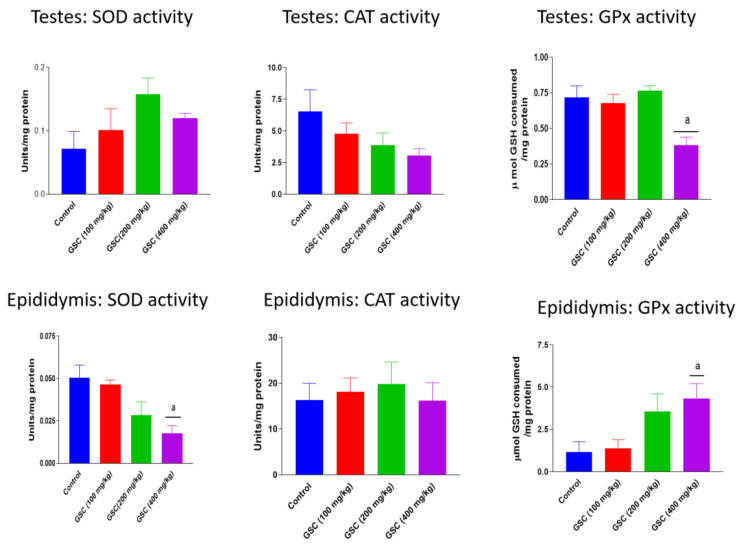
Effect of green-synthesized cellulose (GSC) on antioxidant enzyme activities in testes and epididymis of rats. Values are expressed as mean ±SEM (n = 5). ^a^ Significantly different from control (*p* < 0.05). SOD—superoxide dismutase; CAT—catalase; GPx—glutathione peroxidase.

**Figure 2:**
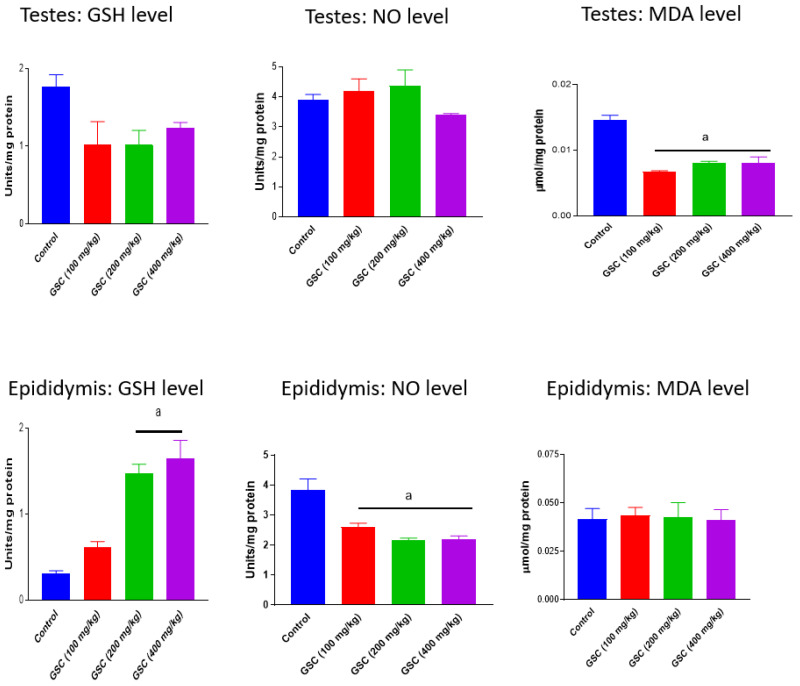
Effect of green-synthesized cellulose (GSC) on oxidative stress parameters in testes and epididymis of rats, Values are expressed as mean ±SEM (n = 5). ^a^ Significantly different from control (*p* < 0.05). GSH—reduced glutathione; MDA—malondialdehyde; NO—nitric oxide.

The activities of testicular and epididymal antioxidant enzymes, SOD, CAT and GPx in rats following 14-day exposure to GSC are presented in Figure 1. Administration of GSC at all treatment doses did not produce any treatment-related effects on testicular SOD and CAT activities as well as epididymal CAT activity. However, high dose GSC (400 mg/kg) caused significant (*p* < 0.05) decreases in testicular GPx (47%) and epididymal SOD (64%) activities and increase in epididymal GPx (270%) activity vis-à-vis the control group. In Figure 2, GSC at 100, 200 and 400 mg/kg significantly (*p* < 0.05) depleted the levels of testicular MDA by 55%, 46% and 47% and epididymal NO by 32%, 44% and 43%, respectively. GSC at the doses of 200 and 400 mg/kg also significantly increased epididymal GSH levels, when compared with the control. Nevertheless, there were no treatment-related changes in testicular GSH and NO levels as well as in epididymal MDA levels in GSC-treated rats when compared with their respective control groups.

### 3.3. Effect of GSC on Histomorphology of the Testes and Epididymis

**Figure 3:**
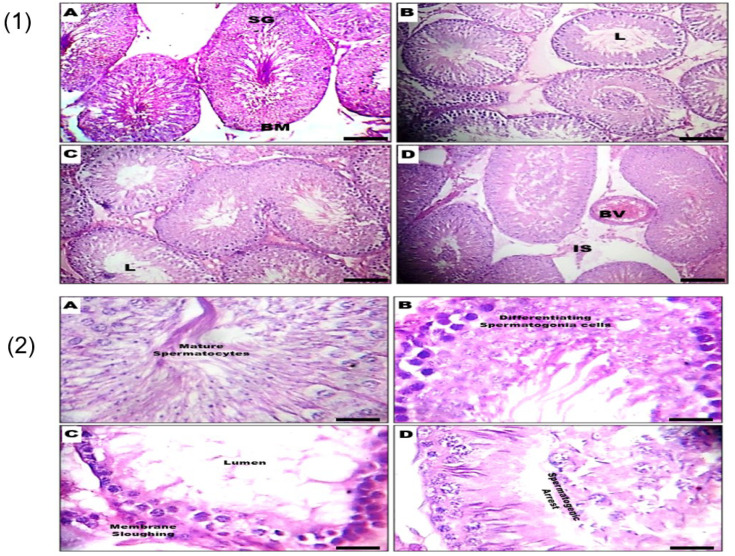
Representative photomicrographs of the testes from control and GSC-treated rats. (**1**) Normal-looking spermatogonium (SG), basement membrane (BM), blood vessel (BV) and the lumen (L) were observed in the (**A**) Control, (**B**) GSC 100-mg/kg, (**C**) GSC 200-mg/kg and (**D**) GSC 400-mg/kg groups (M ×200); (**2**) (**A**) Control and (**B**) GSC 100 mg/kg groups showed normal-looking primary, secondary and tertiary spermatocytes. Treatment-related aberrations such as sloughing of germinal cells, mild stretic spermatogonia and spermatogenic arrest were observed in (**C**) GSC 200-mg/kg and (**D**) GSC 400-mg/kg groups (M ×400).

The histopathology of testicular groups exposed to different doses of GSC did not reveal any discernable dose-dependent severity of lesions at the ×200 magnification. Normal looking spermatogonium (SG), basement membrane (BM), blood vessel (BV) and the lumen (L) were observed across all treatment groups. Similarly, group A (Control) and (B) GSC 100 mg/kg did not reveal any histo–architectural abnormalities at the ×400 magnification as the primary, secondary and tertiary spermatocytes appeared normal looking. However, at the ×400 magnification, treatment-related aberrations such as sloughing of germinal cells, mild stretic spermatogonia and spermatogenic arrest were observed in groups (C) GSC 200 mg/kg and (D) GSC 400 mg/kg (Figure 3).

**Figure 4:**
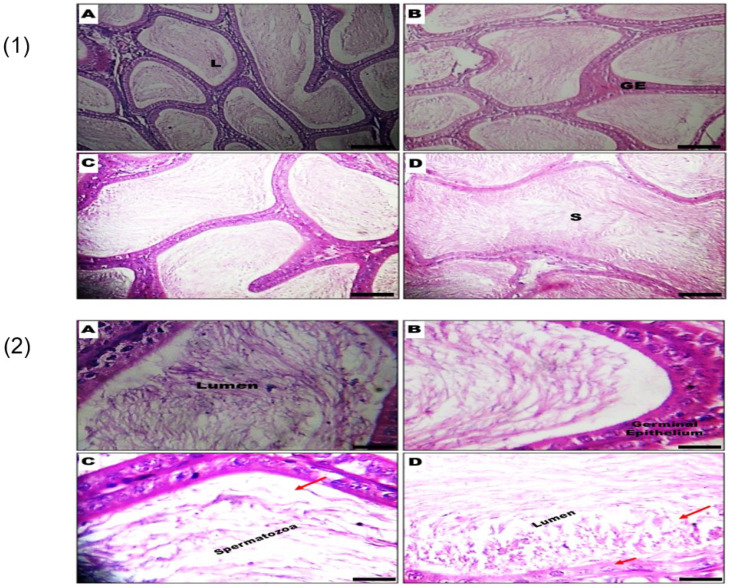
Representative photomicrographs of the epididymis from control and CSC-treated rats. (**1**) Germinal epithelium (GE) appears normal, and there was an abundance of spermatozoa (S) in the lumina (L) of the epididymal duct in (**A**) Control and (**B**) GSC 100-mg/kg groups, while treatment with (**C**) GSC 200 mg/kg and (**D**) GSC 400 mg/kg revealed mild depletion of L content and extravasation of GE in the epididymis (M ×200); (**2**) (**A**) Control and (**B**) GSC 100-mg/kg groups showed normal-looking germinal epithelium (GE) and abundance of spermatozoa (S) in the lumina (L) of the epididymal duct, while treatment with (**C**) GSC 200 mg/kg and (**D**) GSC 400 mg/kg revealed mild depletion of lumina content and chromatolytic changes in the GE (arrowed) (M ×400).

The histopathology of the epididymis at ×200 and ×400 magnifications are presented in Figure 4. In groups A (Control) and (B) GSC 100 mg/kg, no visible lesions were noted at the ×200 magnification, and the germinal epithelium (GE) appeared normal with an abundance of spermatozoa (S) in the lumina (L) of the epididymal duct. Contrariwise, the rats exposed to (C) GSC 200 mg/kg and (D) GSC 400 mg/kg revealed mild depletion of lumina content and extravasation of the GE. Furthermore, at the ×400 magnification, groups A (Control) and (B) GSC 100 mg/kg showed normal-looking germinal epithelium (GE) and abundance of spermatozoa (S) in the lumina (L) of the epididymal duct. In contrast, treatment with (C) GSC 200 mg/kg and (D) GSC 400 mg/kg revealed mild depletion of lumina content and chromatolytic changes in the GE (arrowed) (M ×400).

## 4. Discussion

There is a growing attentiveness in cellulose-based products owing to their vast applications in the food, biomedical and pharmaceutical industries. As such, there are several strategies to diversify the sources of cellulose, aside from conventional sources. The diverse applications of cellulose add another level of complexity arising from increased human exposure; therefore, there is an obligation to assess the health implications of exposure to cellulose. We have chosen the male reproductive organs as the target organs of toxicity following our previous study showing the slight testicular-toxicity of cellulose bionanocomposite [7]. Data obtained from this study indicated that GSC treatment in rats did not elicit changes on absolute weights and organosomatic indices of both testis and epididymis. Since the gross weight and organosomatic indices of the testes mirror the condition of the organ following exposure to xenobiotics, the lack of discernible effect of GSC on the germ cells could be inferred from the data [23].

The testicular and epididymal proteins have been suggested to play key roles during spermatogenesis and sperm maturation. Bjorkgren and Sipila [24] agreed that proteins found in the testis and epididymis is involved in sperm function. In our study, testicular and epididymal total proteins were statistically insignificantly elevated doses of the doses of 100, 200 and 400 mg/kg GSC, an indication that GSC preserved protein-associated functions. Oxidative stress, arising from the lopsided balance of endogenous antioxidant systems and reactive oxygen species, had long been associated with male infertility. The spermatozoa have a fragile antioxidant defense system and are susceptible to oxidative stress and subsequent compromise of sperm integrity and epigenome [25]. In the testis, oxidative stress impairs the steroidogenic capacity of Leydig cells and the differentiation capacity of the germinal epithelium [26]. Furthermore, nitrosative stress alone or in combination with oxidative stress, has been associated with pathologic aftermaths in the male reproductive organs [27,28]. During the initiation of spermatogenesis and the epididymal maturation, the reproductive organs deploy preventive antioxidant enzymes such as SOD, CAT and GPx to counteract internally generated reactive oxygen species [29,30]. In the current study, rats administered with GSC showed differential effects on testicular and epididymal antioxidant enzymes, except CAT. GSC, at all treatment doses, did not disturb CAT activity in both testis and epididymis, lending credence to earlier reports that this enzyme may have limited importance in male reproductive organs [7,31]. In the testis, SOD activity remained unchanged, while epididymal SOD activity decreased at the high dose GSC treatment. A possible explanation is that testicular spermatozoa, protected and nourished by the Sertoli cells, are more resistant to the activities of free radicals in comparison to the epididymal spermatozoa with minimal protection in the lumen [32]. More important, the vulnerability of the testis and epididymis to malondialdehyde, an index of lipid peroxidation, was reversed by the administration of GSC, and this reversal was more obvious in the testis than in the epididymis. The complex interplay between SOD, superoxide anions and nitric oxide in the formation of peroxynitrite has been highlighted in an elegant review article [33]. Typically, SOD ubiquitously present in cellular compartments catalyzes the dismutation of superoxide anions into hydrogen peroxide. Interestingly, nitric oxide, although present at extremely lower concentrations than SOD, has a faster reaction rate with the superoxide anions than SOD. Consequently, basal peroxynitrite is continuously produced, even at physiological conditions, such that induction of SOD activity alone does not completely abrogate its formation. The increase in GPx and GSH levels in the high dose GSC treatment, as observed in this current study, could be a synergistic strategy of the antioxidant molecules to suppress nitric-oxide-mediated peroxynitrite formation in rat epididymal tissue.

Spermatogenesis, in the seminiferous tubules of the testes, involves intricate cell differentiation processes to produce sperm cells. Spermatogonia found in the germinal epithelium could self-renew or differentiate into spermatocytes, which undergo meiosis to generate spermatids. In the final phase of spermatogenesis, the observable features include spermatid elongation, chromatin remodeling and formation of the flagellum and acrosome. Finally, the spermatozoa are released into the lumen of the tubule to continue the maturation in the epididymis [34]. At the ×200 magnifications, this study identified the spermatogonia, basement membrane and the lumen, while the higher magnification of ×400 identified the spermatocytes. Moreover, the use of the higher magnification enabled the identification of treatment-related aberrations such as sloughing of germinal cells, mild stretic spermatogonia and spermatogenic arrest in the testes of rats exposed to GSC (200 mg/kg) and GSC (400 mg/kg). In the epididymis, the control and GSC (100 mg/kg) groups gave similar outcomes at both levels of magnification. However, some magnification-specific histological changes such as extravasation (×200 magnification) and chromatolytic aberrations of the germinal epithelium (×400 magnification) were observed.

## 5. Conclusions

This study presents preliminary data on the effect of GSC on male reproductive histoarchitecture and oxidative stress parameters. In the future, a subchronic study is needed to clarify the effects of this green-synthesized cellulose on reproductive hormones and testicular enzymes. Nevertheless, this study is a giant step toward the safety profiling of GSC as a prelude for future applications.

## Figures and Tables

**Table 1:** Absolute weights, organosomatic indices and protein levels of testes and epididymis in rats administered green-synthesized cellulose (GSC).

	Control	GSC (100 mg/kg)	GSC (200 mg/kg)	GSC (400 mg/kg)
Testes (g)	2.08 + 0.19	2.37 + 0.11	1.96 + 0.17	2.37 + 0.60
Organosomatic index (Testes)	1.22 + 0.06	1.17 + 0.04	1.12 + 0.08	1.14 + 0.20
Epididymis (g)	0.90 + 0.11	0.90 + 0.11	0.87 + 0.07	0.99 + 0.07
Organosomatic index (Epididymis)	0.48 + 0.05	0.46 + 0.01	0.50 + 0.03	0.48 + 0.01
Testicular total protein (g/dL)	3.33 + 0.14	3.63 + 0.37	3.61 + 0.51	4.09 + 0.38
Epididymal total protein (g/dL)	2.38 + 0.01	2.81 + 0.24	3.17 + 0.62	3.23 + 0.48

Data expressed as mean ± SEM of five animals.
